# Kawasaki disease: guidelines of the Italian Society of Pediatrics, part I - definition, epidemiology, etiopathogenesis, clinical expression and management of the acute phase

**DOI:** 10.1186/s13052-018-0536-3

**Published:** 2018-08-30

**Authors:** Alessandra Marchesi, Isabella Tarissi de Jacobis, Donato Rigante, Alessandro Rimini, Walter Malorni, Giovanni Corsello, Grazia Bossi, Sabrina Buonuomo, Fabio Cardinale, Elisabetta Cortis, Fabrizio De Benedetti, Andrea De Zorzi, Marzia Duse, Domenico Del Principe, Rosa Maria Dellepiane, Livio D’Isanto, Maya El Hachem, Susanna Esposito, Fernanda Falcini, Ugo Giordano, Maria Cristina Maggio, Savina Mannarino, Gianluigi Marseglia, Silvana Martino, Giulia Marucci, Rossella Massaro, Christian Pescosolido, Donatella Pietraforte, Maria Cristina Pietrogrande, Patrizia Salice, Aurelio Secinaro, Elisabetta Straface, Alberto Villani

**Affiliations:** 1 Bambino Gesù Children’s Hospital, Rome, Italy, Piazza S. Onofrio n. 4, 00165 Rome, Italy; 2 Fondazione Policlinico Universitario A. Gemelli, Università Cattolica Sacro Cuore, Rome, Italy; 3 Giannina Gaslini Institute, Genoa, Italy; 4 Istituto Superiore di Sanità, Rome, Italy; 5 Università degli Studi di Palermo, Palermo, Italy; 6 Università degli Studi di Pavia, Pavia, Italy; 7 Policlinic Giovanni XXIII, Bari, Italy; 8 ASL Umbria 2, Foligno, Italy; 9 Università degli Studi Sapienza, Rome, Italy; 10 Università degli Studi Tor Vergata, Rome, Italy; 11 Ospedale Maggiore Policlinico, Milan, Italy; 12 Ospedale di Battipaglia, Salerno, Italy; 13 Università degli Studi di Perugia, Perugia, Italy; 14 University of Florence, Florence, Italy; 15 Università degli Studi di Torino, Turin, Italy; 16 Associazione Rari ma Speciali, Rome, Italy

**Keywords:** Kawasaki disease, Coronary artery abnormalities, Intravenous immunoglobulin, Aspirin, Children

## Abstract

The primary purpose of these practical guidelines related to Kawasaki disease (KD) is to contribute to prompt diagnosis and appropriate treatment on the basis of different specialists’ contributions in the field. A set of 40 recommendations is provided, divided in two parts: the first describes the definition of KD, its epidemiology, etiopathogenetic hints, presentation, clinical course and general management, including treatment of the acute phase, through specific 23 recommendations.

Their application is aimed at improving the rate of treatment with intravenous immunoglobulin and the overall potential development of coronary artery abnormalities in KD. Guidelines, however, should not be considered a norm that limits treatment options of pediatricians and practitioners, as treatment modalities other than those recommended may be required as a result of peculiar medical circumstances, patient’s condition, and disease severity or complications.

## Background

In 2008 the first Italian Kawasaki disease (KD) Guidelines were published. During the subsequent years new data have been collected and updated treatment reports have been published.

### Scope

Scope of these revised guidelines is to update evidence on the following topics:

- potential etiopathogenesis of KD;
- definition of clinical signs and symptoms, laboratory, and instrumental tests useful for KD diagnosis;
- efficacy of therapy for KD in the acute phase of the illness.

### Users

These guidelines are directed to pediatricians who work in hospital, family pediatricians, and general practitioners who work with children affected by KD and for families of KD patients.

### Note for users

The clinical management of each patient with KD requires the application of these recommendations based on the peculiar patient’s condition. We are pleased to publish updated diagnostic and therapeutic indications for both medical and paramedical staff as well as the most accurate information for families.

### Sponsorships

No person who participated in the drafting of these guidelines has been sponsored.

### Dissemination

The text has been initially discussed during the Consensus Conference “Kawasaki Disease: Italian Guidelines” in Rome during September 2015. The same text has been rediscussed in the 71st National Congress of the Italian Society of Pediatrics in Rome during June 2015, and finally approved in the 73th National Congress of the Italian Society of Pediatrics in Naples during June 2017.

### Updates

Future updates are planned within the next 5 years, or sooner, if the medical literature will reveal evidence showing that these guidelines have become obsolete.

### Methods

Different experts in general pediatric medicine, cardiology, infectious diseases, rheumatology, immuno-allergology, dermatology, radiology, or biologists experts in cell oxidative stress have participated in writing these guidelines. They have been supported by representatives of family associations. The team has been requested to systematically analyze the most recent literature about KD to define the following evidences dealing with etiopathogenesis, definition of clinical signs and symptoms, laboratory, and instrumental tests for diagnosis, efficacy of therapy with intravenous immunoglobulin and aspirin, efficacy of other therapies in the acute phase of KD.

The basic document was the Italian KD Guidelines, published in 2008 (Marchesi A, et al. Malattia di Kawasaki: Linee Guida italiane. Prospettive in Pediatria. 2008;38:266–83). Additionally, further references from the last 8 years were searched, using PubMed and Cochrane databases.

The following key-words were used: “child”, “Kawasaki disease” or “Kawasaki syndrome”, “coronary arteries aneurysm” and “ectasia”, “echocardiography”, “multi-slide computed tomography”, “angiography”, “intravenous immunoglobulin”, “aspirin”, “corticosteroids”, limiting the search to documents on humans, written in English or Italian. Heterogeneity of the available researches and their low number did not allow to perform a meta-analysis of each item. The recommendations of these guidelines are based on the best evidences available. Stronger recommendations are based on high scientific quality data or, alternatively, on the consensus of experts. Clinical guidelines typically provide evidence levels based on the study design (Table 1) and reported effectiveness (Table 2).

**Table 1:** Level (class) based on study design, defined as follows

class I meta-analyses or systematic reviews from randomized controlled trials class II single randomized controlled trials class III nonrandomized controlled trials class IV retrospective case-control studies class V number of cases without control group class VI opinions of committees of experts and authorities

**Table 2:** Classification (grade) based on effectiveness, defined as follows

grade A highly recommended grade B recommended grade C recommended, but evidence is uncertain grade D non recommended grade E contraindicated	

## Introduction

Nine years have passed since the first announcement of the Italian Guidelines for diagnosis and management of Kawasaki disease (KD) in a national journal, but recently many novel data and publications have become available in relationship with this acute systemic vasculitis of childhood [1]. According to the 2012 “Revised International Chapel Hill Consensus Conference Nomenclature of Vasculitides” [2], KD involves small and medium-sized vessels in each organ and tissue. The first KD cases were observed in Japan in 1962, and described by dr. Tomisaku Kawasaki in the article “*Acute febrile mucocutaneous syndrome with lymphoid involvement with specific desquamation of the finger and the toes in children*” in the 1967 journal *Arerugi* [3]. In general terms, we can consider KD as a self-limited heterogeneous disease with unknown etiology, which mostly affects infants and children under 5 years of age.

The most significant complications in KD are coronary artery aneurysms (CAA), but their overall incidence has been consistently reduced by treatment with intravenous immunoglobulin (IVIG) within 10 days of fever onset [4, 5]. Diagnosis of KD is merely clinic, based on the diagnostic clinical criteria, but may be supported by the results of various blood and instrumental exams. Actually, no clinical findings or tests can be considered specific for KD, and this circumstance makes diagnosis especially challenging. Diagnostic difficulties depend on several causes, such as the different times at which clinical findings might appear, difficult discrimination with other infectious and non-infectious illnesses, protean clinical expression of the disease, occurrence of non-typical clinical findings, incomplete forms of the disease, absence of specific laboratory data, and even association with low acute phase reactants. Concurrently, a prompt recognition of KD is essential as its prognosis depends on the rapidity of treatment decision.

Goal of these guidelines is to recommend the best practice in both diagnosis and management of children with KD, based on the most actual scientific evidence, and improve the overall prognosis of this disease. These guidelines have been created for pediatricians working in hospital, family pediatricians, and general practitioners or nurses managing children affected by KD and for families of KD patients.

### Definition of Kawasaki disease

#### Typical KD

Typical or classic KD is characterized by the presence of ≥5 days of fever and ≥ 4 of the following main clinical features: bilateral non-exudative conjunctivitis, erythema of lips and oral mucosa, changes in the extremities, skin rash, and cervical lymphadenopathy. Cases with defervescence within the fifth day since disease onset should be included. Diagnosis of KD is based on the presence of the above clinical criteria: there are neither typical diagnostic features, nor specific diagnostic tests. These clinical criteria appear within 1–2 weeks, therefore the suspicion of KD may be initially problematic. The most fearful KD complications are CAA, which develop in 15-to-25% of untreated patients, but only in 5% of those treated with IVIG within 10 days following disease onset [6]. The medical literature has recently reported an increasing number of patients with incomplete and atypical KD*.*

#### Incomplete KD

Incomplete KD occurs in patients presenting a typical fever *without* a sufficient number of main clinical criteria, with or without CAA. This kind of KD is frequent in children younger than 12–24 months and should be suspected in every child younger than 6 months affected by fever for more than 7 days and a documented systemic inflammation, without any other possible cause [6].

#### Atypical KD

*Atypical KD* occurs in patients presenting a typical fever and signs or symptoms different from the main KD clinical features (i.e. meningeal inflammation, seizures, facial paralysis, acute abdomen, acute pancreatitis, cholestatic jaundice, arthritis, renal injury, pneumonia, etc.), with or without CAA [6].

##### Recommendation 1

Typical or classic KD is diagnosed if fever ≥5 days associated with ≥4 diagnostic criteria, with or without CAA, or if fever lasts at least for 4 days with ≥4 diagnostic criteria and eventual demonstration of CAA on echocardiography.

(III - A)

##### Recommendation 2

Incomplete KD can be diagnosed when patients with fever for ≥5 days lack a sufficient number of clinical criteria (≤3) to fulfill the previous recommendation, with or without CAA.

(III - A)

##### Recommendation 3

Atypical KD is diagnosed if fever, not otherwise explained, lasting for ≥5 days is associated with classic diagnostic criteria and non-classic manifestations, with or without CAA.

(III - A)

### Epidemiology of Kawasaki disease

Epidemiologic data for KD are mainly available for Asia, Europe and North America, but there are also estimates for Australia, South America, Middle East, along with few data also from Africa [7–11]. The latest Italian data were produced in 1984 [12]. Studies on the spread of the disease have shown considerable differences between different geographical areas (Table 3), with incidence from 3.4 up to 218.6 cases per 100.000 children less than 5 years [7–13]. The overall incidence in Asia is, on average, at least 3–4 times higher in certain countries (Japan, Korea, China, Taiwan) rather than other (India, Thailand); these differences are even more significant in comparison with Europe and USA. It is likely that these differences are due to ethnic factors combined with environmental factors, but the different ability to recognize and report the disease should also be taken into account [11, 14, 15]. In fact, KD incidence in the Japanese population living in Hawaii is similar to those living in Japan [11]. The incidence of KD is different according to gender, as males are more prone to develop the disease, with a M:F ratio of 1,4-1,9:1 emerging from many studies [7–14].

**Table 3:** Incidence rates of Kawasaki disease in Asia, Europe, and North-America

Region	Period	Incidence^a^	Source of data	Reference
Asia				
Japan	2011–2012	243.1–264.8	National survey	Makino et al. 2015 [13]
Japan	2007–2008	215.3–218.6	National survey	Nakamura et al. 2010 [39]
Korea	2009–2011	115.4–134.4	National survey	Kim et al. 2014 [8]
Corea	2006–2008	113.1	National survey	Park et al. 2011 [28]
Taiwan	2003–2006	69.0	National Health System database	Huang et al. 2009 [40]
China				
-Bejing	2004	55.1	Beijing’s Hospitals survey	Du et al. 2007 [41]
-Shanghai	2007	53.3	Pediatric ward survey	Ma et al. 2010 [42]
-Hong Kong	1997–2000	39.0	Retrospective and perspective analysis	Ng et al. 2005 [43]
- Sichuan	2001	9.81	Hospital survey	Li et al. 2008 [44]
India	2007	4.5	Retrospective analysis in Chandigarh, Northern India	Singh et al. 2011 [45]
Thailand	2002	3.4	KD National Register	Durongpisitkul et al. 2006 [46]
North America				
Hawaii	1996–2006	50.4	Hawaii State Inpatient Database	Holman et al. 2010 [47]
Canada	2004–2006	26.2	Clinical records’ Review in Ontario	Lin et al. 2010 [48]
South America				
Chile	2001–2007	6.8	National survey	Borzutzky et al. 2012 [49]
Australia				
Western Australia	1980–1989	2.82	ICD-9 review in Referral Hospital	Saundankar et al. 2014 [9]
	1990–1999	7.96		
	2000–2009	9.34		
Middle- East				
Israel	1996–2009	6.4	National Health System database	Bar-Meir, 2011 [10]
Europe				
United Kingdom	1998–2003	8.4	Hospitals’ data	Harnden et al. 2009 [50]
Ireland	1996–2000	15.2	Ireland’s Hospital In-Patient Enquiry database	Lynch et al. 2003 [51]
Finland	1992	7.2	Recovery register	Salo et al. 1993 [52]
Denmark	1999–2004	4.9	Danish National Hospital Register	Fischer et al. 2007 [53]
Sweden	1990–1992	6.2	Clinical records	Schiller et al. 1995 [54]
France	2005–2006	9.0	Perspective survey in pediatric ward of Northen France	Heuclin et al. 2009 [55]
Italy	1981–1982	14.7	Clinical records’ Review and families’interview in Northen Italy	Tamburlini et al. 1984 [12]

^a^Incidence rates are reported per 100,000 children < 5 years of age, with exclusion of India, for which they are reported per 100,000 children < 15 years of age

Recent epidemiologic data showed an increased incidence of KD during the last decades, both into Western and Asiatic countries [7–9, 11, 13, 16, 17]: this is probably due to an improved ability for practitioners to recognize and diagnose KD, especially in its incomplete form [11, 18]. For instance, in Australia the incidence has increased from 2.82 cases for 100.000 children of age < 5 years during the decade 1980–1989 up to 9.34 cases during the decade 2000–2009 [9]. Due to this improvement, KD has become the leading cause of acquired heart disease in children living in developed countries [1, 11, 14].

With regards to age, KD is mostly observed in children less than 5 years in over 80% of cases [8, 15], with a peak incidence in the first 24 months of life [11, 14]. However, there are also a few reports of KD in neonates, teenagers, and even in adults [8, 14]. Such cases show a tendency to develop CAA [15, 19], probably as a consequence of a late diagnosis.

Several studies demonstrate a seasonality pattern for KD, with a peak incidence in late winter and during the spring-summer period, though this link into different geographical areas is weak [7, 8, 11, 20]. The study of 25 different countries related to a large number of KD patients has shown a significant increase of cases during wintertime in extra-tropical regions of the Boreal hemisphere, with no significant correlation in the Austral hemisphere [21]. A recent theory suggests a link between tropospheric air currents and the epidemic diffusion of KD, due to the transportation of mycotoxins from China and other areas of the Asian continent [22].

The average incidence for atypical and incomplete KD is between 15 and 36%, with a higher distribution at the ends of the age distribution curve (i.e. for children < 1 year and > 5 years) [23, 24].

KD recurrence risk and incidence of familiar forms are well-documented in the Japanese medical literature and also in North-American studies [25–31]. In Japan the risk of illness for a sibling of a patient with KD is increased by 10 times in comparison with the general population [25], and the probability for children with KD to have a sibling with KD is between 0.17 and 1.6% [26–28]. This percentage is increased up to 13% for twins [29]; 50% of familiar cases occur, on average, up to 10 days later than the index case. Furthermore, 0.7% of Japanese cases with KD has at least one parent with a past history of KD [27, 32]. In Japan and Korea the overall recurrence risk is 3% [8, 27, 30], and the main risk factors are age < 3 years and existence of cardiac sequelae [30].

In regards with KD complications, the incidence of CAA is approximately 15–25% in untreated patients and less than 5% in patients treated with IVIG within 10 days after the onset of fever [11, 19, 33]. The risk of developing CAA is greater in children aged < 12 months and > 5 years, in males, and in the recurring forms of KD, as well as in the cases treated too late with IVIG [19, 20, 34]. In particular, infants younger than 6 months can develop CAA up to 65% of cases, even if correctly treated with IVIG [35]. The incidence of IVIG-resistant KD is 15–18% in relationship with the total number of cases and seems to have its main predictor in the presence of very high values ​​of C-reactive protein (CRP) during KD acute phase [34, 36, 37].

Death related to KD is mostly due to cardiac sequelae, both in the short-term, with a peak of mortality between 15 and 45 days after the onset of fever, and in the long-term, even in adulthood: the mortality rate in patients with KD in Japan was more than 1% up to 1974, and has decreased to 0.1 to 0.2% from 1974 to 1993, with a further reduction between 1993 and 2002 to 0.02–0.09% and down to 0.01% in the most recent cases [6, 13, 38, 39]. A Japanese survey in patients who developed KD between 1982 and 1992, followed up until 2009, has documented that those patients without CAA have a standardized mortality rate equivalent to the general population. On the other hand, those patients with cardiovascular involvement have a significantly higher standardized mortality rate [38].

### Etiopathogenesis of Kawasaki disease

The definite etiology of KD is unknown: the identification of etiopathogenic mechanisms behind KD would be essential to develop preventive strategies and focused therapeutic strategies. Analyzing autoptic samples, plasma cells producing IgA have been identified in the wall of arteries of subjects with KD. Moreover, using synthetic IgA, intra-cytoplasmic inclusion bodies with virus-like particles have been found in intact tissue sections from children with KD, specifically in the ciliated epithelium of medium-sized bronchi [56, 57].

#### From the pathophysiological point of view

Arteritis develops around the eighth-ninth day after KD onset, and CAA start with edema formation in the intima and media of arteries and partial rupture of the internal and external elastic lamina. The arterial wall does not support the internal pressure, especially diastolic, and undergoes distension and deformation, leading to the formation of aneurysms. When aneurysms begin to calcify a further pathological distension may develop within 2–3 years [58, 59].

#### From the pathological point of view

There are three phases in KD:

- *Acute phase.* It has a mean duration of 2 weeks and is characterized by a necrotizing vasculitis with a predominance of neutrophils and macrophages [60, 61].
- *Subacute/chronic phase*. It starts after 15 days and is characterized by the presence of lymphocytes, plasma cells and eosinophils, which migrate to the lumen and alter the adventitia.
- *Convalescence phase.* It is characterized by the proliferation of myofibroblasts that form a concentric mass, which progressively obliterates the lumen.

##### Acute phase and innate immunity

The acute phase corresponds to the activation of innate immunity: changes begin from endothelium and progress to the adventitia; necrosis can gradually destroy the adventitia, resulting in ectasia or aneurysm formation, while platelets display a pro-coagulant phenotype in the second week. Further studies have shown that the interaction with activated platelets is necessary for the decision by neutrophils to migrate into the vessel wall or run into cell death with the formation of “extracellular traps” [62–68].

##### Subacute/chronic phase and acquired immunity

The main actors of this phase are macrophages and lymphocytes. The infiltration of these cells starts from the internal elastic membrane or from the media. The resolution of inflammation may be followed by a later stage dominated by macrophages with a different phenotype (myeloid-derived suppressor cells or monocyte-derived dendritic cells), which trigger a particular aspect of acquired immunity, producing different cytokines, and progress to a final phase characterized by proliferation of myofibroblasts and production of strongly pro-fibrotic cytokines (interleukin-4 and interleukin-13). The involvement of innate and acquired immunity in KD is also confirmed by the presumed association with different genetic polymorphisms in different genes (*ITPKC*; *BLK*; *FcGR2A*) with regulatory functions [69–78].

### Presentation and clinical course of Kawasaki disease

#### General signs and symptoms

Signs and symptoms useful to the diagnosis of KD are defined “diagnostic clinical criteria” and are represented by:

- presence of ≥5 days of fever (including cases of therapy-induced defervescence before day 5),
- bilateral non-exudative conjunctivitis,
- erythema of the lips and oral mucosa,
- changes in the extremities,
- skin rash,
- cervical lymphadenopathy [6].

**Fever** is typically high-spiking and remittent. In the absence of appropriate therapy, fever might persist for a mean of 11 days, but it may continue for 3 to 4 weeks and, rarely, even longer. **Bilateral conjunctival injection** usually involves the bulbar conjunctivae, sparing the limbus, the avascular zone around the iris; it is usually painless, and appears immediately following the onset of fever. Mild acute iridocyclitis or anterior uveitis may be noted by slit-lamp; it resolves rapidly and rarely is associated with photophobia or eye pain; it is mild, bilateral, and resolves within 2–8 weeks. **Changes of the lips and oral cavity** include erythema, dryness, fissuring, peeling, cracking, bleeding of lips, “strawberry tongue” with diffuse erythema of the oropharyngeal mucosa. **Rash** is usually **polymorphic** and nonspecific: it may show various forms, and the most common is a diffuse maculopapular eruption, appearing in over 90% of cases, typically within 3–5 days of fever onset. Bullae or vesicles are not seen. Occasionally seen are urticarial exanthema, scarlatiniform rashes, erythroderma, erythema multiform-like rash, micropustular and pustular eruptions [79]. **Erythema of the palms and soles** may sometimes accompanied by painful induration of both hands or feet. Desquamation of fingers and toes usually begins in the periungueal region within 2 to 3 weeks after the onset of fever and may extend to the palms and soles. Desquamation, usually with a “glove finger” pattern, is not KD-specific, because it might appear in some infectious diseases. Deep transverse grooves across the nails (Beau’s lines) may appear approximately 1 to 2 months after the disease onset. In the acute phase, erythema of the perineal region can be observed, where early desquamation may occur: this is a typical sign of KD, recently proposed among the clinical criteria. **Cervical lymphadenopathy** is the least common of KD principal clinical features, usually unilateral, in the anterior cervical triangle, and involving ≥1 lymph node that is > 1,5 cm in diameter, firm, non-fluctuant, not associated with marked erythema of the overlying skin, and nonresponsive to antibiotic therapy.

#### Other clinical findings of Kawasaki disease

As KD is an acute systemic vasculitis, which can affect medium-small arteries, more clinical findings can be present (the most frequent are in italic).

- **Heart**: *coronaritis, pericarditis*, *myocarditis,* endocarditis, arrhythmia, mitral regurgitation/aortic and tricuspidal regurgitation (in the acute phase), aortic dilation (in a later phase), cardiac failure, shock.
- **Vascular system**: Raynaud’s phenomenon, peripheral gangrene.
- **Joints**: arthritis or arthralgia.
- **Nervous System**: *irritability* (a particularly suggestive symptom of KD, but not yet considered as pathognomonic)*, aseptic meningitis*, encephalopathy, seizures, ataxia, sensorineural hearing loss, transient unilateral peripheral facial nerve palsy.
- **Gastrointestinal tube**: *diarrhea, vomiting, abdominal pain,* acute abdomen, hepatic enlargement and jaundice, acute acalculous distention of the gallbladder (hydrops), acute pancreatitis, cholestatic jaundice, retropharyngeal abscesses are described in 3.6% of cases [80, 81].
- **Urinary system**: *aseptic pyuria*, proteinuria, urethritis, testicular swelling.
- **Skin**: erythema and induration at the site of a previous *bacillus Calmette-Guérin* vaccination [82].
- **Respiratory system**: cough, rhinorrea, slow-resolution pneumonia, pulmonary nodules, and lung infiltrates.

#### Clinical course of Kawasaki disease

KD clinical course can be divided into three clinical phases: acute, subacute, and convalescence phase (Table 4).

**Table 4:** Clinical phases of Kawasaki disease

Acute phase (first and second week): - presence of fever and other acute classic signs	
Subacute phase (third and fourth week): - it begins with defervescence and when other acute signs vanish - irritability, anorexia, and conjunctivitis can still be present - digital skin desquamation - thrombocytosis - coronary artery aneurysms might appear - in this phase there is a risk of sudden death	
Convalescence phase (fifth to eighth week): - all disease signs and symptoms disappear - inflammatory indexes return completely to normal - endothelial dysfunction might cause cardiovascular acute events	

#### Differential diagnosis of Kawasaki disease

Table 5 shows the most frequent illnesses to consider in the differential diagnosis with KD, subdivided into infectious and non-infectious. Excluding all possible infectious diseases is recommended. A simultaneous viral or bacterial infection cannot be excluded, and consequently the identification of viral or bacterial agents cannot exclude KD diagnosis and also IVIG treatment. A concomitant bacterial infection must additionally be treated with antibiotics. It is also important to emphasize that a positive response to IVIG does not automatically confirm diagnosis of KD, as other diseases (e.g. sepsis) can be also treated with this same therapy. Finally, coronary artery dilation is not pathognomonic of KD, and indeed it can be present in the acute febrile phase of both infectious (Epstein-Barr virus infection, and Rocky mountain spotted fever) and inflammatory diseases (systemic onset-juvenile idiopathic arthritis, Takayasu disease, polyarteritis nodosa, and systemic lupus erythematosus) [83–88].

**Table 5:** List of illnesses entering in differential diagnosis with Kawasaki disease

Infectious illnesses	Non-infectious illnesses
. viral (rubella, adenovirus, enterovirus, cytomegalovirus, Epstein-Barr virus, parvovirus B19, human herpesvirus 6) . scarlet fever . *Mycoplasma pneumoniae* infection . toxic shock syndrome . staphylococcal scalded skin syndrome . bartonellosis . Rocky mountain spotted fever . tularemia . leptospirosis	. drug hypersensitivity reaction . Stevens-Johnson syndrome . juvenile idiopathic arthritis . polyarteritis nodosa . autoinflammatory syndromes . sarcoidosis . acrodynia

### Cardiovascular complications of Kawasaki disease

The most relevant complications in KD are represented by CAA, and different remodeling phenomena will affect their prognosis. The coronary artery dilation may start as ectasia, slight expansion (up to less than 5 mm in diameter), moderate dilation (from 5 to less than 8 mm) up to giant aneurysms (more than 8 mm in diameter). The majority of coronary artery aneurysms occur in the proximal segments and at the branch level. KD patients with normal coronary arteries or with mild ectasia at 6 weeks since disease onset have an overall good prognosis [6, 89, 90]. On the contrary, patients with persistent aneurysms are at risk of stenosis and/or thrombosis of the same arteries. Giant coronary aneurysms do not revert to a normal morphology (see Tables 7 and 8 for details). The repair of affected vessels occur by wall remodeling without total “restitutio ad integrum”, but with progressive intimal hyperplasia and fibrosis, that lead to stenotic changes of the coronary artery, with risk of thrombosis, myocardial heart attack, and sometimes even sudden death. Rarely new aneurysms appear later in patients with pre-existing aneurysms and, if this occurs, they represent post-stenotic dilations. In rare cases aneurysms can develop in the axillary or celiac arteries. Other different cardiovascular complications may develop less frequently in patients with acute KD, and include myocarditis, pericarditis or pericardial effusion with myopericarditis, valvular insufficiency, and, rarely, arrhythmias. A specific treatment may be required for these manifestations as well as for cardiac dysfunction or heart failure [91, 92] (see part II).

### Other complications of Kawasaki disease

Anemia, hypoalbuminemia, electrolyte imbalance, liver dysfunction, cholecystitis, seizures, diarrhea, vomiting, dehydration, and heart failure might occur and specific treatments may be required. The occurrence of macrophage activation syndrome has also been reported [93–95], while another potential complication is KD shock syndrome [96] (see part II).

### Recurrence of Kawasaki disease

Recurrence of KD ranges from 1.4 to 3% (respectively from the Chinese and Japanese epidemiologic studies available). Often KD symptoms are the same as for the first episode. A longlasting fever, IVIG resistance, elevated AST levels, reduced hemoglobin are all risk factors related to KD recurrence [97]. A recurring KD is associated with a higher risk of development of CAA. Autoinflammatory syndromes should be considered for a comprehensive differential diagnosis in childhood [98].

### Investigations in Kawasaki disease

Diagnosis of KD is based on the sole clinical diagnostic criteria [5, 6], because neither pathognomonic clinical features nor specific diagnostic tests exist. A prompt diagnosis is crucial because prognosis relies on the rapidity of treatment. Consequently, in case of suspected KD, it is important to recommend patient’s hospitalization to perform a thorough evaluation and confirm diagnosis. Unfortunately, laboratory tests are nonspecific, though they can support KD diagnosis (see Table 6) [1, 6] in patients with suggestive clinical features of KD.

**Table 6:** Main laboratory abnormalities in patients with Kawasaki disease

BLOOD CELL COUNT	
white blood cells	↑, maximal elevation of polymorphonuclear cells ↓ rarely
red blood cells	↓, normal mean corpuscular volume
platelets	↑, typically in the II and III week, normalization in 4–8 weeks if ↓ suspect disseminated intravascular dissemination
INFLAMMATORY PARAMETERS	
erythro-sedimentation rate (ESR)	↑, slow normalization
C-reactive protein (CRP)	↑, fast normalization
LIVER FUNCTION TESTS	
transaminases	↑
bilirubinemia	↑
gamma-glutamyl transpeptidase	↑
albuminemia	more severe and prolonged illness if ↓
OTHER LABORATORY TESTS	
urine	white blood cells > 10/high field powered
cerebrospinal fluid	aseptic meningitis (presence of mononuclear cells, normal glucose/proteins)
synovial fluid	purulent-appearing fluid, white blood cells 125.000–300.000/mm^3^, normal glucose level

#### Laboratory findings

Leukocytosis is typical during the acute phase of this disease with predominance of immature and mature granulocytes. Anemia occurs commonly and is normochromic normocytic: it resolves with the resolution of inflammation. Elevation of acute-phase reactants as ESR and CRP is frequent, but sometimes they are only slightly increased. Moreover, ESR is elevated by IVIG therapy, and therefore, a decreased ESR during follow-up should not be used to assess response to treatment with IVIG. Thrombocytosis is characteristic in the second week of the disease, peaking in the third week and normalizing by 4 to 6 weeks after onset in most cases; thrombocytopenia is rare, but may occur in the first 1 to 2 weeks of illness: thrombocytopenia can be a sign of disseminated intravascular coagulation and is a risk factor for the development of coronary artery abnormalities. In patients with arthritis, arthrocentesis typically yields purulent-appearing fluid with a white blood cell count of 125,000 to 300,000 per mm^3^, a normal glucose level and negative culture tests. Mild to moderate elevations in serum transaminases or gamma-glutamyl transpeptidase occur in 40 to 60% of patients, and mild hyperbilirubinemia occurs in 10%. Hypoalbuminemia is common and associated with more severe and more prolonged acute disease. Urinalysis may show pyuria in up to 80% of children. In children who undergo lumbar puncture, 30% demonstrate pleiocytosis with a mononuclear cell predominance, normal glucose levels and generally normal protein levels.

##### Recommendation 5

Laboratory data are nonspecific in KD and can only support diagnosis in patients with suggestive clinical features.

(V - B)

#### Dyslipidemia and risk of atherogenesis in patients with Kawasaki disease

A disrupted lipid metabolism may characterize the acute phase of KD, and finally give a reduction of serum total cholesterol, particularly HDL, and increase of triglyceridemia. Some studies have reported that this altered lipid state might persist over a period of 3 years after KD diagnosis. The effect of dyslipidemia on the cardiovascular risk is not completely clear: while some studies proved early ultrasound signs of reduced elasticity and abnormal carotid intima-media thickness in young adults with history of KD, subsequent studies were not confirmatory [99–102]. The presence of CAA seems also to correlate more significantly with lipid metabolism disorders, whereas this finding remains controversial in patients without CAA [103].

#### Pro-inflammatory conditions and pro-coagulant activity of the peripheral blood in patients with Kawasaki disease

The formation of reactive oxygen and nitrogen species in KD is known to generate a systemic pro-oxidant status in the blood, which might lead to altered red blood cell and platelet homeostasis with propensity to develop a cascade of procoagulant complications and focal endothelial damage [104–106]. Particularly, inflammation associated with systemic pro-oxidant state can play a key role in the pathogenesis and progression of KD. Reactive oxygen and nitrogen species (collectively called RONS) generate a pro-oxidant status in the blood that promote an oxidative and nitrative stress as well as a redox imbalance, leading to altered cell signaling and functions. Whole blood from patients with KD show increased levels of RONS, while scavenger activity of RONS might be significantly decreased during the acute phase of KD. Finally, thrombocytosis might result from a defect of cell death, as suggested by studies about annexin V positive and negative platelets, and the propensity to develop coronary damage may be associated with altered platelet homeostasis [104–106].

#### Echocardiography

Echocardiography should be performed by a cardiologist with experience in the pediatric age, and the coronary artery districts to visualize include left coronary artery, descending coronary artery, circumflex artery, left anterior artery, right coronary artery, and posterior descending coronary artery. The presence of marked perivascular brightness, the absence of the physiological gradual reduction of coronary artery caliber, and a mild ectasia, if isolated, are not indicative of KD; however, these findings could be considered a positive echocardiographic sign when they are all three simultaneously present (expert opinion) to suggest KD diagnosis. Changes in the left ventricular function, presence of mild mitral or aortic regurgitation, and pericardial effusion may be present in the KD acute phase, though they should not be considered sufficient for confirming KD diagnosis [111]. Stenosis and thrombosis might appear in later phases and/or in older patients, when the coronary arteries are more difficult to see on routine ultrasound investigations. Stress echo with the administration of drugs (dobutamine) is now widely used for the diagnosis and follow-up of secondary ischemic heart disease in patients with KD [112, 113].

##### Recommendation 6

Bidimensional and color-Doppler echocardiography and/or associated techniques (tissue-Doppler imaging, 3D echocardiography imaging) are pivotal modalities to perform cardiac assessment of patients with KD, as they are non-invasive repeatable investigations, characterized by both high sensitivity and high specificity.

(V - A)

Criteria for the recognition of KD-related CAA have been defined by the Japanese Ministry of Health, based on the absolute values ​​of coronary arteries size among patients of different age groups [107]. However, in cases that did not meet these criteria it was reported an increased incidence of CAA at different phases of the disease, compared to patients belonging to homogeneous and comparable groups for age and body surface area [108].

The concept of z-score was introduced several years ago to compare the coronary artery diameter to the body surface area and measure the standard deviation from the average in Z units (SD, z-score), using specific nomograms (www.parameterZ.com). This is recommended for the right coronary, left anterior descending, left main coronary arteries, and also for other vascular structures such as the aortic ring and ascending aorta. We define a z -score < 2.5 normal, *viceversa* pathological if ≥2.5, with features of “ectasia” or “aneurysm” depending on the morphological characteristics observed. This standard deviation system should be used in the initial diagnosis of CAA when there is a suspicion of KD, when the selection may be more “coarse” to avoid losing patients that may be at risk in the near future; conversely, size criteria should be used in the follow-up of KD patients, particularly if localized coronary artery injuries have been found (see Table 7) [1, 109, 110].

**Table 7:** Classification of coronary artery aneurysms in the acute phase of Kawasaki disease and severity classification

Dilation: diameter ≥ 2.5 SD	
Small aneurysm: localized dilation showing an inner diameter ≤ 4 mm (in children ≥5 years: the internal diameter of a segment < 1.5 times compared to an adjacent segment) Z-Score ≥ 2.5 to < 5	
Medium aneurysm: aneurysm showing an inner diameter > 4 mm and < 8 mm(in children ≥5 years: the internal diameter of a segment 1.5~ 4 times compared to an adjacent segment) z-score ≥ 5 to < 10	
Giant aneurysm: aneurysm showing an internal diameter ≥ 8 mm(in children ≥5 years: the internal diameter of a segment > 4 times compared to an adjacent segment) z-score ≥ 10	

The severity of Kawasaki disease is classified into the following 5 grades on the basis of findings of echocardiography and selective coronary angiography or other methods (see Table 8) [110].

**Table 8:** Severity classification of coronary artery aneurysms in Kawasaki disease

I. No coronary dilatation: patients with no coronary dilatation including those in the acute phase	
II. Transient coronary dilatation during the acute phase: patients with slight and transient coronary dilatation which typically subsides within 30 days after onset	
III. Regression: patients who still exhibit coronary aneurysms meeting the criteria for dilatation or more severe change on day 30 after onset, despite complete disappearance of changes in the bilateral coronary artery systems during the first year after onset, and who do not meet the criteria for Group V	
IV. Remaining coronary aneurysm: patients in whom unilateral or bilateral coronary aneurysms are detected by coronary angiography in the second year or later and who do not meet the criteria for Group V	
V. Coronary stenotic lesions: patients with coronary stenotic lesions detectable by coronary angiography	
(a) Patients without ischemic findings: patients without ischemic signs/symptoms detectable by laboratory tests or other examinations	
(b) Patients with ischemic findings: patients with ischemic signs/symptoms detectable by laboratory tests or other examinations	

In up to 80% of patients with significant dilation or aneurysms at later echocardiograms some abnormality is evident on the initial echocardiogram obtained in the first 10 days of illness [1]. The largest proportion of patients with coronary artery abnormalities will have dilation only, characterized by luminal measurements outside the normal range, but with a maximal *z-*score < 2.5. Dilation resolves within 4 to 8 weeks in most cases..

##### Recommendation 7

Echocardiogram must be performed in all patients with a diagnosis of KD; then after 2, 4, and 8 weeks since KD onset in the uncomplicated cases, as CAA can be detected in the subsequent weeks after diagnosis.

(VI - B)

##### Recommendation 8

Persistently febrile non-responders KD patients with CAA, impaired left ventricular function, mild/moderate mitral regurgitation or significant pericardial effusion need a more frequent echocardiogram check-up (at least twice per week).

(VI - A)

The presence of marked perivascular brightness, the absence of the physiological gradual reduction of coronary artery caliber and mild ectasia, if isolated, are not indicative of KD. These findings could be considered a positive echocardiographic KD sign when they are all three simultaneously present (expert opinion). Changes in left ventricular function, presence of mild mitral or aortic regurgitation, pericardial effusion may be present in the KD acute stages but should not be considered sufficient for the diagnosis, as they can also be found in other inflammatory disease.

Moreover, pericarditis, mitral insufficiency, impairment of systolic function, indirect signs of inflammation, can be predictive of coronary lesions, which could be non permanent. Actually echocardiographic sensibility and specificity is not so clear in diagnosis of stenosis and thrombi.

These lesions appear usually in later phases and in older patients, when coronary arteries system is more difficult to see.

Echocardiography remains the instrument of choice to identify CAA during the acute phase of KD up to the first 6 weeks after onset. However, computed tomography (CT) or magnetic resonance (MR) angiography can be required for an accurate risk stratification via evaluation of the vascular system, especially in growing children. Advanced cardiovascular imaging techniques for a more specific assessment of cardiovascular sequelae in KD are CT and MR angiography. Multi-slice CT is an established tool for a less-invasive assessment of the cardiovascular system in adults: the diffusion of such a technique for coronary artery studies in children with KD is still limited. This is mainly due to radiation exposure issues, need for general anesthesia, need for beta-blockers (to obtain regular heart rates ideally lower than 65–70 beats per minute), and enhancement contrast infusion. A dual-source CT (DSCT) provides excellent quality images and low radiation doses for diagnosing KD-related CAA in infants and children [114]. Moreover, DSCT is superior to echocardiography in the detection of CAA located in the peripheral parts of vessels [115]. New generation scanners enable a very rapid body CT scan (less than 1 s), permit central/peripheral aneurysm detection, and reduce the need for general anesthesia [116]. The majority of magnetic resonance (MR) angiography studies on KD have been performed using 1.5 Tesla magnets fully equipped for cardiac investigation with a set up for general anesthetic support, using a comprehensive scan protocol which includes functional imaging with breath-hold, ECG-gated, 2D steady-state free precession, contrast-enhanced study for whole body detection of central and peripheral aneurysmal dilations during intravenous infusion of contrast (gadolinium at a dose of 0.1 mmol/kg of body weight), or myocardial perfusion imaging at rest and after pharmacological stress [117–119]. Advanced imaging studies with cardiovascular CT or MR may be reasonable in selected patients to help in the management decisions, as cardiac catheterization in the acute phase of KD has been associated with a greater incidence of adverse vascular events [1]. Conversely, there are limited data regarding cardiac function after the acute phase of KD. Subclinical dysfunctions may be missed when assessing left ventricular systolic function using conventional echocardiography. Recently, speckle-tracking echocardiography is emerging as an effective tool for determining global longitudinal and circumferential strain and myocardial deformation in patients with KD, especially in mid-long term follow-up [120].

##### Recommendation 9

Cardiovascular CT scan, ideally with a DSCT scanner, should be used in patients with KD to:

- confirm CAA (and rule out false positive cases due to coronary artery anomalies or anatomical variants)
- detect middle-distal CAA (not usually seen at routine echocardiograms)
- more accurately define caliber and morphology of CAA
- identify coronary artery thrombosis or occlusions
- evaluate other aneurysms, both central or peripheral, in the entire body
- reveal myocardial ischemia or reassess caliber and morphology of CAAs and better define their treatment.
  (VI - C)

Cardiovascular CT scan in the further follow-up phases can be used to monitor the results of previously treated coronary arteries (e.g., after stenting) (see Part II for details).

##### Recommendation 10

Cardiovascular MR angiography should be used in patients over 8 years with KD to:

- confirm CAA (and rule out false positive cases due to coronary artery anomalies or anatomical variants).

- identify other aneurysmal dilations, either central or peripheral, in the vascular system.

- assess biventricular global/regional systolic function.

- depict any myocardial scar with contrast-enhancement and visualize gross coronary artery anatomy.

(VI - C)

In the further follow-up phases cardiovascular MR angiography can be used to detect areas of inducible myocardial ischemia during pharmacological stress (e.g., adenosine stress perfusion test) (see Part II for details).

### Treatment of Kawasaki disease

In this first part of the Guidelines the only treatment of the acute phase will be discussed.

## Treatment of the acute phase of Kawasaki disease

The main goal of treatment in the acute phase of KD is suppressing the inflammatory reaction and minimizing the risk of developing CAA. A host of data about the administration of intravenous immunoglobulin (IVIG) as initial “*first-line*” treatment in children with KD has confirmed the efficacy and safety of this therapy. In fact, following IVIG introduction since the early 1980s, the overall incidence of CAA is gradually decreased [121]. IVIG should be started before the 7th day of illness: in fact, histological studies have shown that KD arteritis typically develops around the 8-9th day after disease onset. In about 80% of cases, defervescence occurs within 48 h after IVIG infusion.A persistent or reoccurring fever after 48 h since a first administration of IVIG configures the framework of IVIG-resistant KD (approximately 15–20% of patients). Nowadays there is no still effective and universally accepted treatment for these patients, and therefore treatment of these challenging patients will have to be cut out ad hoc. In addition, there is no universally accepted classification system for evaluating the severity of KD or possibility to modulate therapy: many scoring systems have been proposed, but they have shown little sensitivity in the Western populations than in Japanese one [122].

The initial treatment of KD acute phase includes the combined use of IVIG and aspirin (acetylsalicylic acid, ASA).

### Intravenous immunoglobulin (IVIG)

#### Rational

High-dose IVIG is the most effective anti-inflammatory treatment to reduce the risk of CAA in patients with KD [123–126], as confirmed by a systematic Cochrane review [127]. IVIG is derived from human plasma and requires an informed consent before use.

#### Mechanism of action

Since KD causes are unknown, the mechanisms of action of IVIG remain speculative. The hypothesized action mechanisms mainly are anti-inflammatory and mediated by the Fc receptor, B and T cells, and dendritic cells [128–131].

#### Therapeutic indication

IVIG is indicated in the acute phase of typical, incomplete and atypical KD; IVIG should not be administered if fever spontaneously disappears, if CAA are not shown, or if inflammatory markers (ESR and CRP) are within normal limits.

#### Dosage

A dose of 2 g/kg of body weight in a single infusion significantly reduces the incidence of CAA, and is able to suppress both inflammation and fever. The rate of infusion of IVIG varies slightly for different products, though IVIG is usually administered in 12 h in North America and in 24 h in Japan. Currently there is no apparent difference for efficacy among different brands of IVIG.Close monitoring and reduced rate of infusion are necessary during the first 30–60 min in order to reduce the risk of anaphylaxis. During the administration of IVIG, presence of fever or a further rise in temperature do not contraindicate the administration which should not be withdrawn. IVIG should be started within 7 days since KD onset to rapidly reduce inflammation, as in the 8-9th day CAA might appear. One study compared patients treated with IVIG within the 5th day of illness with those who received IVIG between the 6th and 9th day: the groups did not differ in the incidence of resistance to therapy or number of days of hospitalization, though 1 year later those who received IVIG earlier had a lower incidence of coronary artery disease [132]. In addition, we must consider that KD enters a differential diagnosis with many diseases, therefore treatment of suspected cases before the 5th day of fever could intercept patients with other diagnoses. Furthermore, the administration of IVIG is expensive and might be related to even severe, although infrequent, side effects. Treatment before the 5th day of fever should be reserved to exceptional cases of unequivocal diagnosis of KD [133].

#### Effectiveness

IVIG is quite safe and, at present, are the most effective therapy in KD.

#### Side effects

The donated blood is carefully screened to confirm the absence of HBsAg, anti-HCV antibodies, anti-HIV-1/2 and anti-HTLV-1 antibodies, and to verify the absence of nucleic acid from HIV, hepatitis B virus, hepatitis C virus, hepatitis A virus, and human parvovirus B19. Using the existing pharmaceutical production processes, the absence of viruses cannot be determined with 100% certainty, but there have been no reports of viral infections caused by IVIG infusion. Therefore, side effects are rare, and include chills, hypotension, anaphylactic reactions, aseptic meningitis, hemolytic anemia, abnormal liver function tests, jaundice, acute renal failure, thrombocytopenia, and pulmonary edema. Patients should be closely monitored, particularly immediately after the start of IVIG infusion and when increasing the infusion rate. Patients may also develop cardiac dysfunction or acute heart failure, and therefore it is necessary to prevent a sudden increase of circulating blood volume. When using IVIG, it is necessary to consider allergic reactions in response to IVIG in patients with IgA deficiency, the risk of a further deterioration of renal function in patients with a previous kidney damage, thromboembolic events such as cerebral infarction or myocardial infarction in patients with previous or present cardiovascular or cerebral damage [134, 135].

##### Recommendation 11

IVIG must be administered at dose 2 g/kg of body weight in a *single* infusion, within the first 7th day of illness, anyway within the 10th day. Administration should be performed in 12 h if patient’s cardiac function is normal, or in 16–24 h for patients displaying cardiac failure.

(I - A)

##### Recommendation 12

IVIG should also be administered to children presenting after the 10th day of illness in case of:

- persistent fever
- no more fever but aneurysms and ongoing systemic inflammation, as shown by elevation of CRP.

(V- B)

### Aspirin (acetylsalicylic acid, ASA)

#### Rational

The mechanism of action of aspirin differs according to the dosage: medium-high dosages are used for the anti-inflammatory effect in the acute febrile phase of KD, while lower dosages are used for the antiplatelet effect in the subacute phase, when the risk of CAA is higher.

#### Mechanism of action

ASA blocks the synthesis of prostaglandin E2 from arachidonic acid, thereby exerts its anti-inflammatory effect, but also inhibits platelet aggregation by blocking the synthesis of thromboxane A2 by cyclooxygenase.

#### Indications

ASA is approved for all patients with KD.

#### Dosage

Associated with IVIG in the initial treatment, ASA anti-inflammatory dosage varies from 80 to 100 mg/kg/day in the USA to 30–50 mg/kg/day in Japan, subdivided into four doses [136]. Then, 48 h after defervescence, aspirin can be used at an antiplatelet dosage: 3–5 mg/kg/day. The duration of treatment is 6–8 weeks in KD patients without CAA. However, in patients with CAA, antiplatelet therapy is continued until resolution of the lesions or indefinitely in case of persistence.

#### Effectiveness

Many studies do not demonstrate that the anti-inflammatory effect of aspirin reduces the development of CAA; furthermore, recent studies suggest to start immediately with an antiplatelet dosage in the acute phase of KD, in consideration of the procoagulant state of platelets in KD [137, 138].

#### Side effects

The use of ASA at medium-high dosage can be associated with risk of hemorrhage, asthma, liver failure, gastrointestinal ulcers, hives, rash, kidney failure, and loss of appetite. Liver failure is frequent, therefore assessment of transaminase level is mandatory; in case of changes it is necessary to reduce ASA dosage or stop treatment. In case of chickenpox (varicella) and flu the possibility of Reye’s syndrome should be considered, while at low dosage of ASA the occurrence of this syndrome is rare.

##### Recommendation 13

In the KD acute phase ASA must be administered at a daily dosage of 30–50 mg/kg divided into 4 doses until 48 h after disappearance of fever.

(I - A)

##### Recommendation 14

When high-dose ASA is discontinued, low-dose ASA (3-to-5 mg/kg per day) must be started.

(I- A)

In the medical literature there is an ongoing debate about discontinuation of aspirin depending on the size of CAA.

##### Recommendation 15

In patients without CAA low-dose ASA is to be discontinued 8 weeks after KD onset. In children who develop CAA low-dose ASA may be continued until the resolution of vascular lesions or indefinitely in case of its persistence.

(V - B)

Attempting to identify which KD patients should be unresponsive to treatment with IVIG or predicting the ones who might display a significant risk of developing cardiologic sequels are two issues of outstanding priority. Many efforts have been made to discriminate as soon as possible the cohort of IVIG nonresponder patients in order to undertake a more aggressive treatment since KD onset: different scores have considered multiple factors, such as patient’s age, sex, duration of disease, white blood cell count, hematocrit, platelet count, C-reactive protein, transaminases, total bilirubin, albumin, sodium, however none of them has shown high sensitivity in Caucasian populations [139].

High-risk KD patients are younger than 12 months, show elevated CRP, elevated aminotransferase level, hypoalbuminemia, severe anemia at disease onset and might display early development of CAA, signs of macrophage activation syndrome or shock.

##### Recommendation 16

High-risk KD patients should receive initial therapy with IVIG + ASA + corticosteroid (1 pulse of intravenous methylprednisolone: 30 mg/kg/day as a single administration or intravenous prednisone: 2 mg/kg/day, then tapered orally).

(I - B)

#### Vaccines: The potential interactions with IVIG and ASA

The analysis of KD cases reported by the Vaccine Adverse Event Reporting System (VAERS) of USA up to 2007 showed that the incidence of this disease in children who received all registered vaccines was similar or even less than the incidence expected on the basis of the known epidemiology of KD in Italy. On the contrary, the administration of vaccines is associated with a lower frequency of occurrence of KD at least for the 42 days following vaccine administration without differences among the various preparations [140]. A recent study, reporting data of 1.721.186 children between 0 and 6 years old, followed by a long period of time, shows that in KD patients the frequency of administration of vaccines in the 42 days preceding the onset of the syndrome was lower than controls non-vaccinated [141].

KD treatment with IVIG does not significantly interfere with all inactivated vaccines, oral attenuated live virus vaccines (rotavirus, typhoid vaccination), live virus nasal vaccines (flu), bacillus Calmette-Guérin (BCG) vaccine, and yellow fever vaccine. Consequently, these vaccines can be administered concurrently with the administration of IVIG or at any time after administration of IVIG in KD. It is usually recommended to wait 10 months before administration of vaccine against measles, mumps, rubella (MMR), varicella (V), and MMRV vaccines, as this could interfere with the immune response to MMR, V and MPRV antigens, which may result in a reduced immunogenicity [142].

In KD patients who are receiving ASA flu vaccination is strongly recommended for the known relationship between the influenza virus infection and the development of Reye’s syndrome in children and adolescents treated with salicylates [143]; for the same reason it is discussed about the administration of V vaccine in children with KD on ASA prophylaxis. Pharmaceutical companies producing vaccines containing V (as a monovalent preparation or as MPRV formulation) indicate on the package that subjects who receive salicylates should discontinue these medications for 6 weeks following the administration of this vaccine [144]. This attitude is not shared by several scientific authorities for the lack of data indicating an actual risk of the vaccine in these patients. Some experts suggest deciding whether or not to vaccinate against chickenpox based on an accurate assessment of the risk of vaccination and those of the disease in the individual patient. If you decide to vaccinate KD children on ASA with V or MPRV vaccines, ASA might be replaced by clopidogrel: its dosage is 1 mg/kg/day in a single dose (up to a maximum of 75 mg/day in children > 2 years, or 0.2 mg/kg of body weight in children < 24 months). Vaccination against V or MPRV could be given after 48 h of stopping ASA, and ASA should be restarted by suspending clopidogrel 6 weeks after vaccination [145]. If a KD patient requires treatment with biologic drugs, it should be assumed that there may be problems of reduced immunogenicity and safety of vaccines [146–149]. As for the live-virus attenuated vaccines, even if there is no evidence of adverse events in the few studies available, it is believed that further studies are needed to confirm their safety; therefore, it is important that the patient receives two doses of MMR vaccine and is adequately protected even against varicella in case of no history of a natural disease for at least 1 month before the start of biological drugs [150].

##### Recommendation 17

As the administration of every type of vaccine does not increase the risk of reappearance of KD, vaccinations are to be administered in KD patients.

(III - A)

##### Recommendation 18

All inactivated vaccines, rotavirus, oral typhoid, intranasal anti-flu, BCG vaccines and the vaccine against yellow fever may be administered concurrently with the administration of IVIG or at any time after administration of IVIG in KD patients.

(V - A)

##### Recommendation 19

The MMR, V and MPRV vaccines should be administered 10 months after the administration of IVIG in KD patients to avoid a reduced immune response as result of the specific high antibody titer against the antigens contained in IVIG.

(VI - B)

##### Recommendation 20

Influenza vaccination is highly recommended in KD patients assuming ASA due to the increased risk of Reye’s syndrome.

(III - A)

##### Recommendation 21

If a KD patient assuming ASA has to be vaccinated with V or MPRV vaccines, ASA can be replaced by clopidogrel because of a theoretical risk of Reye’s syndrome associated with the attenuated varicella-zoster virus contained in these vaccines. Its dosage is 1 mg/kg/day in a daily administration, up to a maximum 75 mg/day (adult dose), or 0.2 mg/kg of body weight in children < 24 months. Additionally, V or MPRV vaccination can be given after 48 h of stopping ASA, and ASA can be restarted after suspending clopidogrel 6 weeks after vaccination.

(VI - C)

##### Recommendation 22

It is recommended to administer all inactivated vaccines following the routine schedule in patients with KD receiving biological drugs.

(V - B)

##### Recommendation 23

Attenuated live virus vaccines should be administered 1 month before beginning therapy with biologics in patients with KD, but they are not recommended during treatment with biologics.

(VI - C)

## Discussion

Not applicable.

## Conclusion

Goal of these guidelines is to recommend the best practice in both diagnosis and management of children with KD, based on the most actual scientific evidence, and improve the overall prognosis of this disease. In this first part of guidelines we carried out hypothesis about etiology of KD, because the identification of its etiopathogenic mechanisms would be essential to develop preventive and focused therapeutic strategies. We improved definition of incomplete and atypical KD, avoiding the loss of potential patients. We tried also to evaluate as dyslipidemia, proinflammatory conditions and procoagulant activity might act as cardiovascular risk factors in KD. Concerning instrumental techniques, we strongly emphasized the suggestion of implementing the use of standard deviation system of z-score in echocardiography, to avoid losing patients during the acute phase of KD. We explained other advanced cardiovascular imaging techniques, i.e. computed tomography (CT) or magnetic resonance (MR) angiography, which can be required for an accurate risk stratification of the vascular system. Concerning therapies, we revised timing if IVIG infusion as well as dosages of aspirin. Attempting to identify which KD patients should be unresponsive to treatment with IVIG or predicting the ones who might display cardiologic sequels, we introduced a stratification in low and high-risk patients with different therapeutic indications. Finally we investigated the potential interactions between vaccines and both IVIG and ASA.

## Abbreviation

ASA: Acetylsalicylic acid
BCG: Bacillus Calmette-Guérin
CAA: Coronary artery aneurysms
CRP: C-reactive protein
CT: Computed tomography
ESR: Erythrocyte sedimentation rate
HbsAg: Hepatitis B surface antigen
HCV: Hepatitis C virus
HIV: Human Immunodeficiency virus
HTLV-1: Human T-cell leukemia virus type 1
IVIG: Intravenous immunoglobulin
KD: Kawasaki disease
MMR: Measles, mumps, rubella
MMRV: Measles, mumps, rubella, varicella
MR: Magnetic resonance
V: Varicella
